# Pathologic Femur Fracture through Osteoid Osteoma after Radiofrequency Ablation: Case Report and Review of the Literature

**DOI:** 10.1155/2021/5560037

**Published:** 2021-07-28

**Authors:** Nathan B. Rogers, Brennan P. Roper, Alfred A. Mansour

**Affiliations:** McGovern Medical School at UTHealth Houston, Houston, TX, USA

## Abstract

This is a case report of a 4-year-old girl who sustained a femoral shaft fracture 2 weeks after radiofrequency ablation of an osteoid osteoma. The fracture occurred after a relatively low-energy impact, jumping off the second to last step of a staircase. The pathologic fracture was successfully treated with closed reduction and spica casting, with full return to activities. Cases have been reported in the literature of femoral shaft fractures in older patients after radiofrequency ablation, but all are farther out than 2 weeks and none in patients as young as 4 years.

## 1. Introduction

Osteoid osteomas (OOs) are benign tumors that can occur anywhere in the axial or appendicular skeleton; however, they are more common in the diaphysis or metaphysis of the lower extremity long bones [1, 2]. OOs are the third most common benign bone tumor, most commonly occurring in patients aged 10–20 years, and are 2-4 times more common in males than females [1]. They are small, usually <1 cm, osteoblastic, slow-growing bone tumors that cause characteristic aching night pain. This pain is thought to occur due to prostaglandins produced by osteoblasts in the tumor itself that affect small, unmyelinated nerve fibers surrounding the OO nidus [1, 3, 4].

Nonoperative treatment consists of nonsteroidal anti-inflammatory drugs (NSAIDs) and salicylates (aspirin) [1, 3]. However, if medications fail to relieve the pain associated with an OO or if the patient requires more expedient definitive treatment, surgical management may be indicated [5–8]. Surgery originally consisted of en bloc resection of the OO [1]. This gradually evolved to less invasive methods such as computed tomography (CT) and fluoroscopic-guided percutaneous resection by drilling and curettage [9]. These methods left a large cortical defect and were eventually supplanted by an even less invasive treatment, radiofrequency ablation (RFA). RFA of OOs has now become the mainstay of surgical management [5, 6, 10, 11].

RFA utilizes CT or fluoroscopic guidance to localize the nidus of the OO. Through a small hole drilled in the cortex, a needle probe is inserted and placed directly on the tumor nidus. Current is run through the probe, generating heat, causing local tissue necrosis, and killing tumor cells. The radius of local treatment for a standard single electrode RFA probe tip is approximately 16 mm [11]. Since it was first introduced in 1992 by Rosenthal, RFA has been reliably successful in treatment of OOs requiring surgical management. Success rates for complete pain relief and symptom resolution range between 75 and 100% [7, 10–15].

Complications after RFA range from minor, such as local skin irritation or wound complications, to more severe, such as tumor recurrence or fracture. Skin irritation has been commonly reported, especially in tibial lesions. These are usually superficial and require only local wound care. Some wounds persist however and may necessitate surgical debridement [10, 11, 16, 17]. Recurrence of tumors is not an infrequent complication, occurring in 7-10% of cases [3, 11]. Treatment failure is thought to be due to incomplete ablation of the OO nidus, and repeat RFA has been successful in complete resolution [11]. Other complications, such as intraosseous broken probe tips, intramuscular hematomas, and postprocedural numbness, have also been reported [7, 17].

An uncommon but severe complication after RFA for OO is fracture, specifically of the subtrochanteric/proximal femur [13, 15, 18]. Only 3 reports have been documented, all in patients ranging from 12 to 20 years old, and all occurred greater than 2 months after RFA. To our knowledge, there has been no previous report of a patient as young as that presented here nor a patient who has sustained a fracture in such a short time frame after RFA.

## 2. Case Report

**Figure 1:**
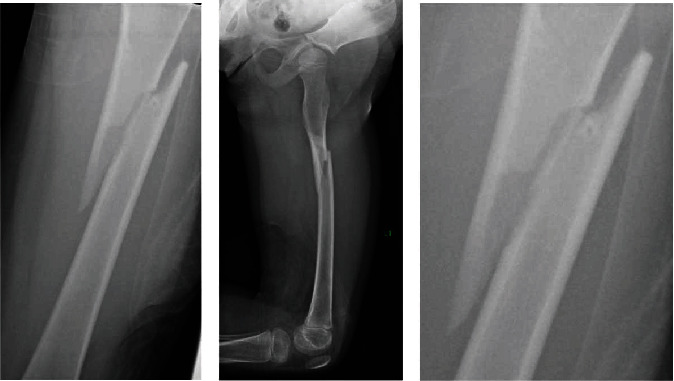
Left femur fracture around prior osteoid osteoma. The patient was 2 weeks s/p RFA of her OO.

A 4-year-old girl was transferred to our institution for a left femoral shaft fracture. She was descending stairs and jumped from the second to last step, landing on her left leg. She noticed an immediate snap and sharp, stabbing pain. Radiographs identified a long oblique proximal femoral shaft fracture (Figure 1).

Upon closer examination, an area in the lateral cortex of the femur at the level of the fracture was visualized showing a small nidus surrounded by sclerotic bone. Further questioning revealed that the patient had a history of an osteoid osteoma in the same femur.

**Figure 2:**
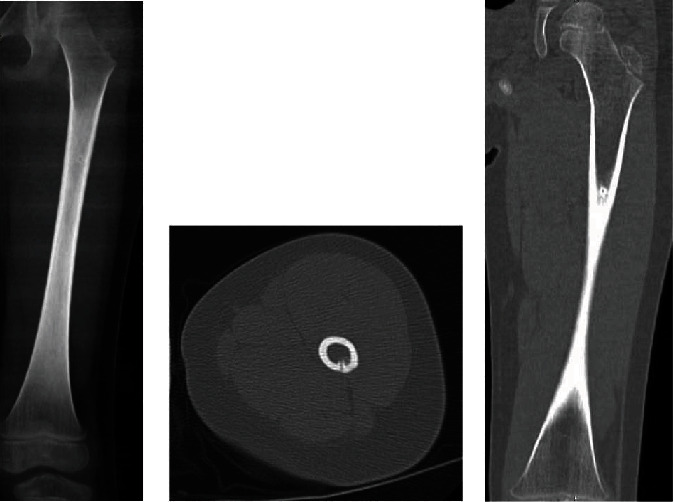
X-ray and CT images of OO 3 months prior to fracture, 2.5 months prior to RFA.

She had originally experienced night pain for 6 months and had a CT 3 months prior, demonstrating the OO (Figure 2).

**Figure 3:**
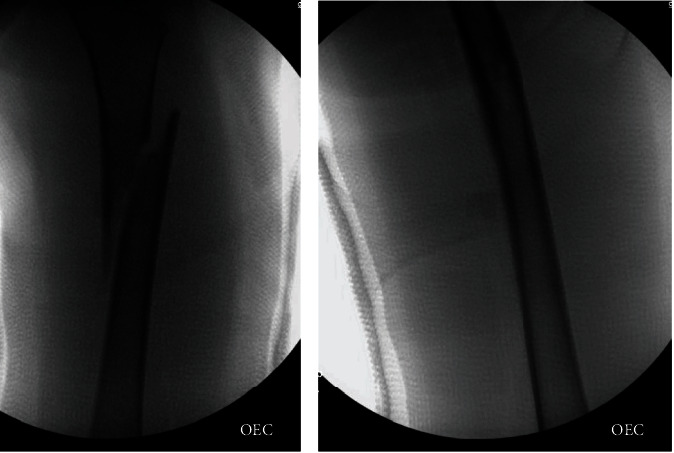
Intraoperative fluoroscopy after closed reduction and spica cast application.

Treatment consisting of NSAIDs and activity modification was attempted for many months, but failed to provide symptom relief. She underwent RFA at an outside institution 2 weeks prior to presentation and was doing well, until the aforementioned injury. She was treated promptly with closed reduction and 1 ½ hip spica cast application (Figure 3).

She tolerated the procedure well and was discharged the following day without incident.

**Figure 4:**
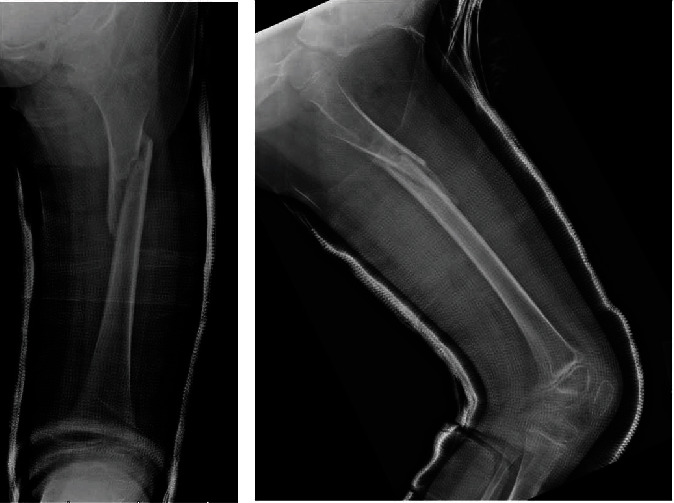
Healing X-rays from clinic 5 weeks after spica cast application.

Her postoperative course was routine and repeat radiographs continually showed a healing fracture with extensive callus formation (Figure 4).

**Figure 5:**
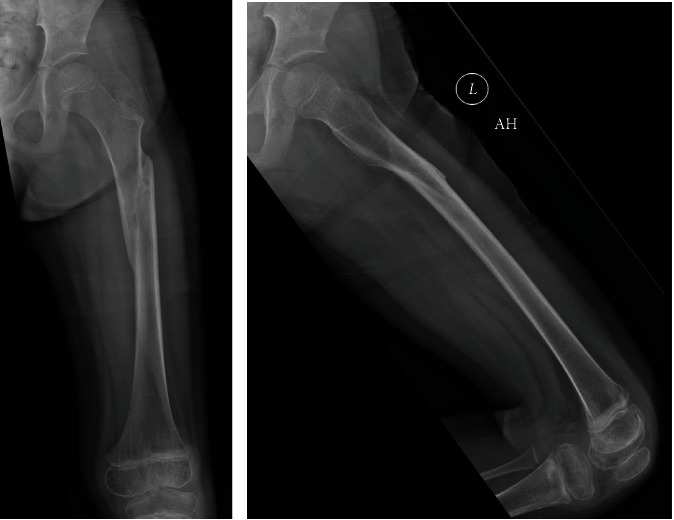
Healed X-rays from clinic 10 weeks after spica casting.

Her spica cast was removed at 5 weeks postoperatively and she was kept non-weight-bearing for a total of 6 weeks, followed by progressive return to weight-bearing as tolerated. Final consolidation was found to occur at around 10 weeks after surgery, and she was released to weight-bearing and activity as tolerated. She had no signs of the prior osteoid osteoma and extensive fracture callus formation (Figure 5).

She was last seen in clinic at 12-month follow-up where she was doing well and had no residual leg pain.

## 3. Discussion

Femur fracture after RFA of an OO is a major complication that has been reported sparsely in the literature. The proximal femur bone morphology demonstrates differing mechanical properties depending on the direction of the applied load. Briefly, the anatomic axis of the femur is in approximately 7 degrees of valgus compared to the mechanical axis. This causes eccentric loading of the proximal femur, with compressive loads on the medial cortex of the proximal femoral shaft and the inferior femoral neck. Concurrently, the lateral cortex of the proximal femoral shaft and superior femoral neck experience tensile loads, placing these anatomic locations at a biomechanical disadvantage. These anisotropic properties offer an explanation for the weakening of this anatomic region after RFA, increasing susceptibility to fracture [13, 18].

Prior to the use of RFA for OO, wide excision or percutaneous resection was performed when nonoperative treatment methods failed [1]. This was performed with en bloc resection of the OO or CT localization and resection using a 7 mm toothed drill bit to remove the lesion. Assoun et al. reported on a 19-year-old patient who returned to vigorous activity one month after resection of a femoral OO and sustained a femoral shaft fracture [9]. The exact anatomic location of the femur fracture was not noted. However, with a 7 mm hole, presumably in the lateral cortex of the proximal or middiaphysis, one could understand why this patient fractured only one month after surgical resection.

To access and ablate an OO in the proximal femur, RFA occurs through a small drill hole in the lateral femoral cortex, followed by introduction of the catheter tip that contains a radiofrequency generator [10, 11]. The lateral femur is used for its ease of access and to avoid the medial anatomic structures. Once the catheter tip is located at the site of the OO, radiofrequency ablation occurs at a temperature of 90 degrees Celsius for approximately 5-6 minutes [5, 11]. This increased temperature not only kills the cells of the OO through thermal necrosis but also the surrounding cells for an approximately 1 cm radius. The combination of local cellular necrosis weakening the surrounding bone as well as a cortical breach in the lateral cortex offers a reasonable explanation for the occurrence of post-RFA subtrochanteric and proximal femur diaphyseal fractures.

There have been few case reports of fractures occurring through an OO after undergoing RFA in the pediatric and young adult population. Earhart et al. reported on the results of RFA for OO in 21 children from 2004 to 2010 [13]. They had a follow-up on 17/21 (81%) at an average of 17 months, and no patient had residual or postprocedural pain at the site of their original OO. Two complications were reported, one superficial skin burn managed with local wound care and one late subtrochanteric femur fracture. The fracture occurred through the OO site of a 12-year-old boy while wrestling with a friend 9 weeks after RFA. They discuss the fact that the necrotic bone in the surrounding region after RFA is unable to repair the microscopic damage of daily activity, and therefore, an increased fracture risk exists.

Bonicoli et al. reported a 17-year-old male who had RFA of an OO 10 years prior to injury [15]. He sustained a subtrochanteric femur fracture during a long jump at the same anatomic location as his OO. Mazzawi et al. reported a 20-year-old male military recruit who sustained a midshaft femur fracture while running approximately 1 year after undergoing RFA on his OO [18]. Both authors concluded that activity restrictions should be followed and patients should refrain from high-impact activities after RFA due to weaker sclerotic bone, although the duration of this recommendation remains unclear.

## 4. Conclusion

To our knowledge, this is the youngest patient reported in the literature to sustain a femoral shaft fracture after RFA of an OO. This also represents the shortest time frame from RFA to fracture reported. Due to the varied time frame of pathologic fracture post-RFA, recommendations for postprocedural management can be difficult. We agree with prior recommendations for 6 weeks of decreased activity after RFA; however, further study is needed to elucidate the optimal time frame for restrictions. Additionally, we did not appreciate any delayed healing through the RFA site. We aim to increase awareness of post-RFA pathologic fracture, especially in the proximal femur.

## Data Availability

The case report references used to support the findings of this study can be found in PubMed. Any specific requests for the individual patient data have been recorded in the manuscript. Any questions or requests can be directed to Nathan Rogers at nathan.b.rogers@uth.tmc.edu.
